# Denture Hygiene Knowledge, Practices, and Stated Willingness to Use a Probiotic-Based Denture Cleanser Among Removable-Prosthesis Wearers at a Saudi University Dental Hospital: A Cross-Sectional Study

**DOI:** 10.3390/healthcare14203466

**Published:** 2026-10-15

**Authors:** Rua S. Babaier, Sarah M. Almuhayya, Waled M. Alshhrani, Hassan M. Alsaleh, Ghosoun Alfudily, Sara A. Alfadda

**Affiliations:** 1 Department of Prosthetic Dental Sciences, College of Dentistry, King Saud University, Riyadh 11545, Saudi Arabia; walshhrani@ksu.edu.sa (W.M.A.); saralfadda@ksu.edu.sa (S.A.A.); 2 Department of Clinical Laboratory Sciences, College of Applied Medical Sciences, King Saud University, Riyadh 12372, Saudi Arabia; 3 College of Dentistry, King Saud University, Riyadh 11545, Saudi Arabia; hassan.moh.alsaleh@gmail.com (H.M.A.); ghosounalfudily@gmail.com (G.A.); 4 Faculty of Dentistry, University of Toronto, Toronto, ON M5G 1G6, Canada

**Keywords:** denture hygiene, oral probiotics, oral microbiome, denture stomatitis, knowledge–practice gap, removable prosthesis, health care, public health, Saudi Arabia

## Abstract

**Highlights:**

**What are the main findings?**

- Although 97% of removable-prosthesis wearers considered denture cleaning important, only one in five used denture-cleansing tablets, and neither self-reported cleaning frequency nor knowledge predicted objective denture cleanliness.
- Awareness of the oral microbiome and of oral probiotics was below 20%; half of the participants said they would try a probiotic-based denture cleanser and a third were undecided, although these responses were unstable on retest.

**What are the implications of the main findings?**

- The gap between valuing denture hygiene and achieving clean dentures supports evidence-based denture-care education for patients and practitioners.
- Stated willingness to try such a product supports acceptability, feasibility, and adherence studies of probiotic-based denture cleansers before any claim of clinical benefit.

**Abstract:**

**Background/Objectives:** Many denture wearers rely on ineffective home-care methods, and oral probiotics are an emerging hygiene adjunct of unclear acceptability. This study evaluated denture hygiene knowledge, self-reported practices, oral microbiome/probiotic awareness, and stated willingness to use a probiotic-based denture cleanser among Saudi removable-prosthesis wearers, and their association with objective clinical findings. **Methods:** In this single-center cross-sectional study, 107 removable-prosthesis wearers attending King Saud University Dental Hospital were recruited by convenience sampling. Participants completed a purpose-designed bilingual questionnaire assessed for content validity and test–retest reliability, and underwent a standardized clinical examination by one calibrated prosthodontist. A composite probiotic/microbiome knowledge score (0–4) was derived from four items. Outcomes included the Denture Cleanliness Index (DCI), biofilm accumulation, retention, stability, fit, and denture stomatitis. Non-parametric tests and multivariable logistic regression were used. **Results:** Mean age was 58.6 years (SD 13.2) and 58.9% were women. Although 97.2% considered denture cleaning important, evidence-based methods were used infrequently (cleansing tablets, 20.6%). Awareness of the oral microbiome (17.8%) and of oral probiotics (18.7%) was low, and 61.7% scored 0 or 1 of 4 on the composite knowledge score (mean 1.19). Self-reported cleaning frequency and knowledge score did not correlate with the DCI or biofilm scores. In exploratory models, female gender was associated with lower odds of both a low knowledge score and poor denture cleanliness. Overall, 51.4% said they would try a probiotic-based denture cleanser, 35.5% answered “Maybe”, and 13.1% were unwilling. Responses to this item were unstable on retest (κw = 0.169), so these figures are preliminary. **Conclusions:** In this single-center sample, perceived importance of denture hygiene was not matched by evidence-based cleaning practice, and self-reported hygiene did not correspond to objective denture cleanliness. Awareness of oral probiotics was low, while stated willingness to try a probiotic-based denture cleanser was common. These findings indicate a need for evidence-based denture-hygiene education and for clinical evaluation of probiotic-based denture cleansers; they do not establish the effectiveness or acceptability of such products.

## 1. Introduction

Removable dentures remain widely used to restore function and aesthetics in completely and partially edentulous patients. However, denture wearers may experience discomfort, denture stomatitis, halitosis, and temporomandibular disorders [1,2]. These complications can substantially impair quality of life, particularly among older adults, and are influenced by the type, fit, usage habits, and oral hygiene practices of dentures [3,4,5].

Microbial biofilm accumulation on denture surfaces is a major contributor to denture-associated infections, including denture stomatitis and oral candidiasis [6,7]. The risk increases with overnight denture wear and ineffective cleaning. In Saudi Arabia, studies report a notable prevalence of denture stomatitis, reaching 23.4% in some regions, with significant contributing factors including prolonged denture use, poor hygiene, and *Candida* colonization [8,9].

Several methods are available for denture cleaning, including mechanical brushing, chemical soaking, and antimicrobial rinsing. However, many denture wearers have limited knowledge of effective hygiene practices or do not follow them consistently [10,11]. In Eastern Saudi Arabia, 82.7% of denture wearers reported cleaning their dentures daily. Nevertheless, only 19.3% used denture-cleansing tablets, whereas 41% used potentially abrasive toothpaste [12]. That study did not include a clinical examination and therefore could not determine whether self-reported practices corresponded to objective denture cleanliness. In addition, attendance at maintenance visits is also poor. One study reported that 73.6% of denture wearers did not return for follow-up care [13].

Educational gaps also affect dental practitioners: in a survey of 200 dental professionals in India, 85% reported that denture cleansers received no or only minimal coverage in their dental curriculum [14]. Comparable data on Saudi practitioners have not been reported, as the available Saudi studies have assessed denture wearers rather than the clinicians who advise them [12,15]. This gap highlights the need to strengthen denture-care education for both patients and the professionals who deliver it.

In recent years, oral probiotics have gained attention as an adjunct to conventional denture care. Probiotics are live microorganisms that confer health benefits when administered in adequate amounts [16]. Selected strains of *Lactobacillus* and *Streptococcus* may help modulate the oral microbiome, inhibit *Candida* growth, and reduce biofilm formation on denture surfaces [17,18,19].

Clinical studies suggest that probiotics may reduce *Candida* colonization and symptoms of denture stomatitis among institutionalized and immunocompromised populations [20,21,22]. However, direct comparisons with established antifungal therapies remain limited. Evidence that probiotics provide a safer or more sustainable alternative is also insufficient.

Moreover, this evidence comes almost entirely from probiotic products delivered to the patient rather than to the prosthesis. Those studies administered probiotic lozenges, tablets, or probiotic-supplemented yogurt and measured *Candida* counts or stomatitis severity in the mouth. A probiotic-based denture cleanser is a different proposition, because the product would act on biofilm present on an acrylic surface outside the mouth, and no clinical trial of such a formulation has been published. Patient-delivered probiotic products therefore establish biological plausibility, not clinical efficacy, for probiotic-based denture hygiene, and acceptability is accordingly the appropriate first question.

This study therefore combines a bilingual questionnaire with a standardized clinical examination in the same participants, allowing self-reported hygiene to be compared directly with the measured condition of the prosthesis, and it reports denture wearers’ awareness of oral probiotics and their willingness to use a probiotic-based denture cleanser, which, to our knowledge, have not previously been examined in Saudi Arabia.

The primary objective was to determine whether self-reported denture hygiene knowledge and cleaning practices correspond to objectively assessed denture cleanliness and biofilm accumulation among removable prosthesis wearers attending the King Saud University Dental Hospital in Riyadh, Saudi Arabia. Secondary objectives were to describe awareness of the oral microbiome and oral probiotics and willingness to try a probiotic-based denture cleanser, and to compare clinical denture and oral findings across prosthesis types. Analyses of sociodemographic correlates and of the association between prior probiotic awareness and clinical outcomes were exploratory.

The null hypotheses were:

**H1** **.** *Self-reported denture hygiene knowledge and cleaning practices are not associated with objective denture cleanliness or biofilm accumulation.*

**H2** **.** *Denture cleanliness, biofilm accumulation, retention, stability, fit, and denture stomatitis do not differ among wearers of complete dentures (CD), metal-framework removable partial dentures (RPD-M), and acrylic removable partial dentures (RPD-A).*

Sociodemographic correlates of the knowledge score and of willingness, and the association between prior probiotic awareness and clinical findings, were examined as exploratory analyses without prespecified hypotheses.

## 2. Materials and Methods

### 2.1. Study Design and Participants

This cross-sectional clinical study was conducted at the Prosthodontic Clinics of the College of Dentistry, King Saud University, Riyadh, Saudi Arabia. Ethical approval was obtained from the King Saud University Institutional Review Board (IRB Research Project No. E-25-9941, 15 July 2025), together with the necessary approval from the College of Dentistry Research Center (CDRC No. FR 0740). The study followed the principles of the Declaration of Helsinki. Written informed consent was obtained from all participants before enrollment.

The required sample size was estimated for a cross-sectional prevalence study using the Raosoft sample size calculator and informed by comparable studies [21,23,24]. Assuming an expected prevalence of 50%, a 95% confidence level, and a 10% margin of error, the minimum required sample size was 96 participants. A total of 107 removable prosthesis wearers who fulfilled the eligibility criteria were recruited. No power analysis was performed for the multivariable models, which are therefore interpreted as exploratory (Section 2.8).

Participants were recruited using convenience sampling from patients attending undergraduate, postgraduate, and specialist prosthodontic clinics at the Dental University Hospital. Potential participants were identified through SALUD, the electronic dental record system (Dentsply Sirona, Dublin, Ireland), by screening patients who had been seen for removable prosthodontic treatment within the preceding 5 years, irrespective of how long they had been wearing their current prosthesis. Recruitment was conducted between November 2025 and March 2026.

Participants were eligible if they were aged 18 years or older, living independently, able to provide informed consent, and currently using at least one removable dental prosthesis regularly. Patients were excluded if they had a systemic condition likely to alter oral mucosal or salivary status, defined a priori as uncontrolled diabetes mellitus (HbA1c > 7%, the glycemic target recommended for most non-pregnant adults [25]), immunosuppression (current chemotherapy, radiotherapy to the head and neck, long-term systemic corticosteroid or other immunosuppressive therapy, or untreated HIV infection), pregnancy or breastfeeding, or cognitive or physical impairment limiting routine oral and denture care.

Of 301 electronic records screened, 240 patients met the eligibility criteria and were contacted by telephone. Ninety-eight could not be reached after three attempts and 25 declined, leaving 117 who consented and attended the study visit. Ten were excluded on arrival (withdrawal of consent, or attendance without the prosthesis because it had been forgotten or lost), giving a final sample of 107 participants (response rate 107/240, 44.6%; cooperation rate among those reached 117/142, 82.4%). The recruitment and inclusion flow are shown in Figure 1.

Those who agreed to participate were scheduled for a single clinical visit lasting approximately 15–20 min. Participants were instructed not to clean their removable prostheses for at least 12 h before the appointment [26]. Adherence to this instruction was confirmed verbally with each participant at the start of the visit, and participants who reported having cleaned the prosthesis were rescheduled, so that all examined prostheses met this condition. The study visit consisted of completion of the study questionnaire (Section 2.2) followed by a standardized clinical examination of the removable prosthesis and oral tissues (Section 2.5).

Participants received an oral and denture health check-up as part of the visit. Partially edentulous participants were offered referral for professional oral hygiene care when indicated, while participants requiring replacement of their removable prosthesis were referred to the appropriate prosthodontic clinic.

### 2.2. Questionnaire

A structured questionnaire was available in Arabic and English and comprised five principal sections (Supplementary File S2).

The first section collected sociodemographic and general health information, including age, gender, nationality, marital status, education, health-related educational background, employment status, relevant medical conditions and medications, antibiotic use during the preceding 3 months, recent respiratory infection, smoking status, duration of current denture use, and frequency of dental attendance.

The second section assessed knowledge and awareness, including the source of denture-care information, perceived importance of denture cleaning, awareness of specialized denture-cleaning products, awareness of the oral microbiome and oral probiotics, perceptions regarding potentially harmful oral microorganisms, and knowledge of probiotic dietary sources.

The third section assessed denture hygiene and related behaviors, including denture-wearing habits, denture adhesive use, frequency and methods of denture cleaning, use of denture-care products, previous probiotic use, and consumption of probiotic-containing yogurt.

The fourth section recorded self-reported denture-related problems, including previous oral fungal infection, bad breath, discomfort, redness, or irritation associated with the current prosthesis.

The fifth section evaluated attitudes toward probiotic-based denture cleansers, including willingness to try a probiotic-based denture cleanser and the participant’s preferred product form. Willingness was assessed for a hypothetical product; no formulation, brand, cost or treatment regimen was shown or described to participants (Table 1).

A composite probiotic/microbiome knowledge score was created from four questionnaire items: awareness of the oral microbiome, knowledge that oral microorganisms may be harmful, awareness of oral probiotics, and knowledge that yogurt contains probiotics. Each designated positive knowledge response was assigned 1 point, producing a total score ranging from 0 to 4, with higher scores indicating greater knowledge. The four items were weighted equally because there was no empirical or validated basis for differential weighting. The composite was intended as a descriptive index rather than a validated psychometric scale; it was used descriptively and as an ordinal covariate, and each component item is also reported separately (Table 2).

The questionnaire was developed in English and translated into Arabic by a bilingual prosthodontist (R.S.B.). Both language versions were reviewed by the external expert panel (Section 2.3) for conceptual equivalence, clarity, and cultural appropriateness, and the final version was pilot-tested before the main study. Formal back-translation was not performed.

### 2.3. Questionnaire Content Validity

Content validity of the questionnaire was evaluated before the main study by four independent expert consultants, none of whom were members of the research team, selected for specialist expertise in prosthodontics or oral microbiology and at least ten years of relevant experience. Each expert reviewed the bilingual questionnaire and independently rated every item for relevance, clarity, and simplicity using a 4-point scale, where higher scores represented greater content appropriateness.

Item-level Content Validity Indices (I-CVI) were calculated as the proportion of experts rating each item as 3 or 4. Scale-level Content Validity Indices using the averaging approach (S-CVI/Ave) were calculated as the mean of the item-level indices for each dimension. An I-CVI of ≥ 0.78 and an S-CVI/Ave of ≥ 0.90 were considered acceptable. Items with lower individual ratings were reviewed for conceptual and clinical relevance before retention in the final questionnaire.

### 2.4. Questionnaire Test–Retest Reliability

Before commencing the main study, test–retest reliability was assessed in 14 participants who completed the questionnaire twice, with an interval of approximately 2 weeks between administrations. The same 14 participants served as the pilot sample for both language versions. This sub-study served as a feasibility exercise rather than as formal psychometric validation. The resulting estimates therefore have wide confidence intervals, and agreement statistics are attenuated for items with highly skewed response distributions; both constraints are acknowledged in Section 4.5.

Reliability statistics were selected according to the measurement scale of each variable. Continuous variables were evaluated using the intraclass correlation coefficient ICC(2,1), absolute agreement, with 95% confidence intervals. Quadratic weighted Cohen’s kappa (κw) was used for ordinal variables, and Cohen’s kappa (κ) for nominal and binary variables.

### 2.5. Clinical Examination

The clinical examination was conducted using a standardized checklist (Supplementary File S3) that was followed consistently for each study participant to assess the removable prostheses and associated oral conditions. Prostheses were assessed separately for the maxillary and mandibular arches.

The recorded prosthetic variables included prosthesis classification and type, denture base material, abutment status, where applicable, condition of remaining teeth, retention, stability, and fit. Retention, stability, and fit were each rated on ascending ordinal scales, based on classification schemes described by Kapur [27] and Cosme et al. [28] and applied in comparable recent removable-prosthesis hygiene research [29], in which higher scores indicated better denture quality (retention: 1 = no retention to 4 = good; stability: 1 = no stability to 4 = good; fit: 1 = ill-fitting/fractured to 3 = well-fit).

For the principal prosthesis-type analyses, removable prostheses were categorized as complete dentures (CD), acrylic removable partial dentures (RPD-A), or metal-framework removable partial dentures (RPD-M). No overdenture wearers were identified in the final study sample. Because a substantial proportion of participants wore prostheses of differing type or condition in the upper and lower arches, and because the two arches from a given participant cannot be treated as statistically independent observations, the upper (maxillary) arch was designated as the single representative arch per participant for the prosthesis-type comparisons in Table 3, preserving one independent observation per patient.

For denture hygiene assessment, each removable prosthesis was removed, gently rinsed under running tap water, and air-dried before examination. Denture biofilm accumulation was graded using an ordinal clinical scale, as follows (1 = none to 4 = heavy). Denture cleanliness was evaluated using the Denture Cleanliness Index (DCI) according to the amount of deposits on the fitting surface [30,31], as follows (0 = no visible deposits to 4 = deposits covering most of the fitting surface). Where participants wore prostheses in both arches, the worse (higher) of the two arch-level DCI and biofilm scores was used to represent participant-level denture hygiene status, consistent with the worst-finding representative-scoring principle used in established dental epidemiological indices, such as the WHO Community Periodontal Index of Treatment Needs, which scores each examined region according to its most severe finding [32].

The oral mucosa was examined under standardized clinical illumination with the aid of a tongue depressor. Denture stomatitis was assessed according to Newton’s classification [33], and clinical signs suggestive of oral fungal infection were recorded [7]. Halitosis was recorded as present or absent on the basis of the examiner’s judgment of detectable breath odor during the examination [6]. No standardized organoleptic scale, fixed examiner distance or instrumental measurement was used, so this finding is reported descriptively only and the limitation is noted in Section 4.5.

Salivary status was assessed clinically as normal, reduced, or dry mouth. In addition, salivary flow was measured using a modified Schirmer-type method [34]: a graduated absorbent strip (Diagnostic Schirmer Tear Strips, Desertcart, KSA) was placed on the floor of the mouth for 3 min and the wetted length was recorded in millimeters. Because the strip has a maximum length of 35 mm, values were censored at this ceiling and no validated cut-off for normal flow was applied; the measurement is therefore reported descriptively and was not used in inferential analyses. The qualitative clinical assessment of salivary status is reported separately.

All clinical examinations were performed by a single prosthodontist (R.S.B.) to standardize assessment across participants.

### 2.6. Intra-Examiner Reliability

Intra-examiner reliability of the clinical assessment was evaluated before the main study. Eight participants were examined twice by the same prosthodontist (R.S.B.), with an interval of approximately 2 weeks between examinations. This sample was intended to calibrate the examiner rather than to provide a precise reliability estimate, and the same constraints apply (Section 4.5).

Quadratic weighted Cohen’s kappa was used to evaluate agreement for ordinal clinical variables, unweighted Cohen’s kappa for nominal or binary variables, and ICC(2,1) with 95% confidence intervals for continuous measurements.

### 2.7. Data Management

Questionnaire responses and clinical observations were initially recorded on paper-based case report forms. Completed forms were reviewed for completeness and subsequently transcribed into a secure electronic database.

Each participant was assigned a unique study identification number. Personal identifiers were not included in the analytical dataset, and study data were stored on a secure password-protected local or institutional drive accessible only to authorized members of the research team.

### 2.8. Statistical Analysis

The survey dataset included 107 participants. One participant had no recorded clinical examination data and was therefore retained in survey-based analyses but excluded from analyses involving clinical variables, resulting in a clinical sample of 106 participants. Missing observations were not imputed; an available-case analysis was used, and the sample size was reported for each analysis.

Statistical analyses were performed using R statistical software (version 4.5.1, 13 June 2025; R Foundation for Statistical Computing, Vienna, Austria), with the gtsummary, rstatix, MASS, DescTools, broom, dplyr, tidyr, forcats, ggplot2, patchwork, scales, flextable, and officer packages. Categorical variables were summarized using frequencies and percentages. Continuous variables were described using means and standard deviations and, where distributions were non-normal, medians and interquartile ranges.

Because the principal study outcomes, including the composite knowledge score, DCI, and biofilm accumulation score, were ordinal or non-normally distributed, non-parametric statistical methods were used for inferential analyses. All statistical tests were two-sided, and statistical significance was set at *p* < 0.05.

Differences in the composite probiotic/microbiome knowledge score across sociodemographic groups were evaluated using the Kruskal–Wallis test. Where a significant overall difference was identified, Dunn post hoc pairwise comparisons with Bonferroni adjustment were performed. Associations between knowledge score and continuous or ordinal variables, including age and education level, were examined using Spearman rank correlation.

For multivariable analysis, a lower knowledge score was defined as a composite score of 0–1, compared with scores of 2–4; this threshold was selected based on the observed distribution of the composite score in this sample (median 1, interquartile range 0–2) rather than an externally validated instrument cutoff. Predictors of a lower knowledge score were evaluated using binary logistic regression, and adjusted odds ratios with 95% confidence intervals were reported.

Clinical outcomes were compared across the three prosthesis groups (CD, RPD-M, and RPD-A) using the upper arch as the representative observation per participant (see Section 2.5). DCI and biofilm accumulation scores were compared using the Kruskal–Wallis test. Categorical clinical outcomes, including denture retention, stability, fit, and denture stomatitis, were evaluated using Fisher’s exact tests because of sparse cells. Monte Carlo simulation with 10,000 resamples was used for Fisher’s exact tests where appropriate. Where an omnibus Fisher’s exact test was significant, pairwise Fisher’s exact tests with Bonferroni adjustment were performed.

To investigate knowledge–practice concordance, Spearman rank correlations were calculated between denture-cleaning frequency, the composite knowledge score, worst-arch DCI score, and worst-arch biofilm accumulation. Poor denture cleanliness was defined a priori as a worst-arch DCI ≥ 3, following the published classification of DCI scores of 2 or less as acceptable denture hygiene [31].

The association between denture-cleaning tablet use and poor DCI was examined using Fisher’s exact test. A multivariable binary logistic regression model was subsequently fitted with poor DCI as the dependent outcome; denture-cleaning frequency and knowledge score were entered as the primary explanatory variables, with adjustment for age, gender, and education. Adjusted odds ratios and 95% confidence intervals were reported.

The association between previous awareness of oral probiotics and willingness to try a probiotic-based denture cleanser was assessed using Fisher’s exact test. Willingness was treated as an ordinal outcome ordered as No < Maybe < Yes and further analyzed using ordinal logistic regression. The multivariable model included probiotic awareness, education level, gender, age, yogurt-consumption frequency, and presence of denture stomatitis. Adjusted odds ratios and 95% confidence intervals were reported.

Exploratory comparisons of denture stomatitis, worst-arch DCI, and worst-arch biofilm accumulation according to probiotic awareness were performed using Fisher’s exact or Kruskal–Wallis tests, as appropriate.

Events per variable (EPV) were calculated for each multivariable model: 40 events for four predictors in the knowledge-score model (EPV 10.0), 25 events for five predictors in the poor-cleanliness model (EPV 5.0), and 13 observations in the smallest response category for ten predictors in the willingness model. Models with fewer than ten events per variable are reported as exploratory and their confidence intervals interpreted as wide. Multicollinearity was assessed using variance inflation factors, calibration of the binary models using the Hosmer–Lemeshow test, and the proportional-odds assumption of the ordinal model using the Brant test.

Sensitivity analyses were performed to test the measurement choices described in Section 2.5. The knowledge–practice correlations and the poor-cleanliness model were repeated using the maxillary arch alone and the participant mean of the two arch scores, and the prosthesis-type comparisons were repeated in the mandibular arch. In addition, the composite knowledge score was analyzed as a continuous outcome using ordinal and linear regression with the same covariates, as an alternative to the dichotomized specification.

No family-wise correction was applied across the secondary and exploratory analyses; the probability of at least one false-positive result is therefore inflated, and these findings are reported as hypothesis-generating. Bonferroni adjustment was applied only to post hoc pairwise comparisons following a significant Kruskal–Wallis or Fisher’s exact test.

## 3. Results

### 3.1. Questionnaire Validity and Reliability

Across the 30 rated items, S-CVI/Ave was 0.967 for relevance, 0.958 for clarity, and 0.975 for simplicity. Most items scored I-CVI = 1.00; seven fell below 0.78 on at least one dimension (all 0.75, except clarity of the oral-microbiome item at 0.50). Because the panel had four experts, a single rating below 3 gives an I-CVI of 0.75, so the 0.78 threshold effectively required unanimous agreement. Nine of the twelve sub-threshold ratings came from one expert, and six of the seven items scored 1.00 on at least one dimension (Table S1). All seven items were retained after the research team reviewed them against the study objectives, and their wording was simplified before use. The oral-microbiome item still had to name the term, because it asks whether participants have heard of it.

Test–retest agreement across 41 analysable items (the item on perceived importance of cleaning was excluded for zero within-item variance) was excellent for continuous variables (age ICC(2,1) = 1.000, 95% CI 0.999–1.000; age of youngest child 0.977, 0.916–0.993) and ranged from slight to almost perfect for categorical items: 11 items (26.8%) almost perfect, 11 (26.8%) substantial, 8 (19.5%) moderate, and 11 (26.8%) fair or slight. Agreement was highest for behavioral and demographic items (denture cleaning frequency κw = 0.904; educational level 0.883; smoking status 0.877) and lowest for attitudinal items (willingness to try a probiotic cleanser κw = 0.169) and for low-prevalence cleaning-method items, where high observed agreement was offset by restricted marginal distributions. This slight agreement means that responses to the willingness item were not stable over a two-week interval, so willingness findings are interpreted as a provisional reaction to an unfamiliar product concept rather than a stable preference (Section 4.5).

Of 27 clinical variables, 19 (70.4%) showed almost perfect or excellent agreement, 6 (22.2%) substantial, and 2 (7.4%) moderate. For the principal outcomes (upper/lower arch), κw was 0.662/0.615 for DCI, 0.851/0.800 for biofilm accumulation, 0.800/0.702 for retention, 0.833/0.571 for stability, and 0.833/0.682 for fit; denture stomatitis reached 0.875 and salivary flow rate ICC(2,1) = 0.875 (0.553–0.977). Oral fungal signs yielded κw = 0.000 despite 87.5% observed agreement, reflecting near-zero prevalence rather than unreliable assessment.

### 3.2. Participant Characteristics

A total of 107 wearers of removable prostheses completed the questionnaire and were included in the survey-based analyses (Table 1). One participant had no recorded clinical examination data and was therefore excluded from clinical analyses, resulting in a clinical sample of 106 participants.

The mean participant age was 58.6 years (SD 13.2), with a median of 60 years (IQR 50–68). Women represented 58.9% of the sample, and 72.9% were Saudi nationals. Secondary education was the most frequently reported educational level (31.1%), followed by university education (30.2%). Nearly one-third of participants (29.0%) reported a health-related educational background.

Only 33.6% of participants reported no systemic medical condition. Diabetes mellitus was reported by 15.0%, hypertension by 5.6%, and both diabetes and hypertension by 7.5%. Most participants were never-smokers (86.0%).

More than half of the participants (51.4%) had been wearing their current denture for less than 1 year. Approximately 61.7% attended a dentist at least once every 12 months, while 29.0% attended only when a complaint arose. Verbal instruction from a dentist was the main source of denture-care information for 83.2% of participants, while 10.3% reported never having received denture-care information. Continuous denture wear, including night-time use, was reported by 29.0%.

### 3.3. Denture Hygiene Knowledge and Self-Reported Practices

Almost all participants (97.2%) considered denture cleaning important (Table 2), and 57.0% were aware of specialized denture-cleaning products. However, the reported cleaning methods showed substantial variation. Toothpaste and a toothbrush were used by 41.1% of participants, water alone by 34.6%, and denture-cleansing tablets by only 20.6%. Soap with a toothbrush was reported by 18.7% and soaking of dentures in mouthwash by 14.0% (Figure 2).

Most participants reported frequent denture cleaning: 59.8% cleaned after every meal and 29.0% cleaned once daily at bedtime. Despite this high reported frequency, objective cleanliness outcomes were not significantly correlated with self-reported cleaning frequency.

### 3.4. Oral Microbiome and Probiotic Awareness

Awareness of the oral microbiome and oral probiotics was low (Table 2). Only 17.8% of participants had heard of the oral microbiome, 18.7% were aware of oral probiotics, and 39.3% knew that yogurt may contain probiotics. Previous use of a probiotic product was reported by 16.8%.

The mean composite probiotic/microbiome knowledge score was 1.19 (SD 1.05) out of 4, with a median of 1.0 (IQR 0–2). In total, 61.7% scored 0 or 1; this sample-derived cutoff was used to define a lower knowledge score for the regression analysis. Only one participant achieved the maximum score of 4.

Smoking status was associated with the composite knowledge score (H(2) = 6.038, *p* = 0.049), and post hoc Dunn testing showed lower scores in current smokers than in never-smokers after Bonferroni adjustment (*p* = 0.048). Both results are borderline and rest on small subgroups (92 never-smokers, 9 current smokers, 6 ex-smokers), so this association is unstable and is not carried into the Abstract or Conclusions. Age and education level were not significantly correlated with knowledge score.

In the multivariable binary logistic regression model, female gender was independently associated with lower odds of a knowledge score of 0–1 (adjusted OR 0.29; 95% CI 0.10–0.76; *p* = 0.016). Age, education level, and nationality were not significant independent predictors. When the composite score was analyzed as a continuous outcome with the same covariates, the association with female gender was in the same direction but did not reach significance (ordinal OR 2.01; 95% CI 0.90–4.49; *p* = 0.088), indicating that this finding depends in part on the dichotomized specification. This model included 40 events for four predictors (EPV 10.0), with no evidence of multicollinearity (all variance inflation factors ≤ 1.3) and adequate calibration (Hosmer–Lemeshow *p* = 0.93).

### 3.5. Clinical Denture and Oral Findings

In the lower arch (n = 89), 35 participants wore complete dentures, 30 wore acrylic RPDs, and 24 wore metal-framework RPDs. In the upper arch (n = 88), 50 participants wore complete dentures, 24 wore acrylic RPDs, and 14 wore metal-framework RPDs (Table 3). Of the 106 participants examined clinically, 72 had both arches assessed, 16 the maxillary arch only and 17 the mandibular arch only, and one had no prosthesis assessment recorded (Figure 1). The 18 participants without a maxillary assessment are therefore not represented in Table 3. Worst-arch DCI was available for 104 participants, because one further participant had no DCI recorded.

Median DCI scores were similar among CD, RPD-M, and RPD-A wearers, with a median score of 1.0 in each group (*p* = 0.855). Biofilm accumulation also did not differ significantly among the prosthesis types (*p* = 0.536). Denture retention and fit were not significantly different between groups. In contrast, denture stability differed significantly by prosthesis type (omnibus *p* = 0.038), with sufficient stability observed in 69.2% of RPD-M wearers compared with 46.0% of CD wearers and 29.2% of RPD-A wearers; however, pairwise post hoc comparisons did not reach significance after Bonferroni correction (pairwise Fisher’s exact tests; adjusted *p* = 0.076 for RPD-A versus RPD-M, 0.229 for CD versus RPD-M and 0.497 for CD versus RPD-A), so this finding should be interpreted as a group-level signal rather than confirmed pairwise superiority of any one prosthesis type. Repeating these comparisons in the mandibular arch gave the same pattern, with no significant difference in DCI (*p* = 0.059) or biofilm accumulation (*p* = 0.203) among prosthesis types.

Denture stomatitis was absent in 85.8% of participants, while 11.3% had mild and 2.8% had moderate stomatitis. Clinical fungal signs were uncommon, occurring in 1.9% of participants. Reduced salivary flow was identified clinically in 42.5%, while clinically detected halitosis was present in 3.8%. The modified Schirmer measurement, reported separately from this clinical assessment, was recorded in 106 participants, with a median of 35 mm (IQR 35–35; range 15–35); 85 participants (80.2%) reached the 35 mm ceiling of the strip and 3 (2.8%) recorded values below 25 mm, so the measure was uninformative in this sample and is reported descriptively only.

### 3.6. Knowledge–Practice Concordance and Objective Denture Cleanliness

No significant correlations were observed between cleaning frequency and worst-arch DCI (ρ = −0.058, *p* = 0.559), knowledge score and worst-arch DCI (ρ = 0.125, *p* = 0.207), knowledge score and worst-arch biofilm (ρ = 0.054, *p* = 0.586), or cleaning frequency and worst-arch biofilm (ρ = −0.110, *p* = 0.266). Of the other available correlates, older age was associated with greater biofilm accumulation (ρ = 0.225, *p* = 0.022) and, borderline, with a higher DCI score (ρ = 0.191, *p* = 0.053), whereas duration of denture wear, frequency of denture wear, cleaning method (water alone, toothpaste, or cleansing tablet) and denture adhesive use were not associated with either measure (all *p* ≥ 0.10). The correlations were unchanged when the maxillary arch alone or the mean of both arch scores was used in place of the worst arch (all *p* > 0.2).

Use of denture-cleansing tablets was not associated with poor DCI (worst-arch DCI ≥ 3; Fisher’s exact *p* = 0.578). In the adjusted logistic regression model, cleaning frequency and knowledge score were not significant predictors of poor DCI. Female gender was associated with lower odds of poor DCI (adjusted OR 0.19; 95% CI 0.06–0.57; *p* = 0.004), while age and education were not significant (Table 4). This model included 103 participants, of whom 25 met the definition of poor cleanliness (5.0 events per variable); it is therefore reported as exploratory. Calibration was adequate (Hosmer–Lemeshow *p* = 0.22) and there was no evidence of multicollinearity (all variance inflation factors ≤ 1.3). When poor cleanliness was defined from the maxillary arch alone or from the mean of both arches, female gender remained associated with lower odds of poor cleanliness (OR 0.18, *p* = 0.027 and OR 0.29, *p* = 0.054, respectively), and neither cleaning frequency nor knowledge score became significant. The direction of the gender association was consistent, but its size varied between definitions and it lost conventional significance in one, so the estimate should be regarded as unstable.

### 3.7. Stated Willingness to Use a Probiotic-Based Denture Cleanser

Overall, 51.4% of participants indicated that they would try a probiotic-based denture cleanser, 35.5% responded “Maybe”, and 13.1% were unwilling (Figure 3a). Because a “Maybe” response indicates uncertainty rather than intention, these categories are reported separately throughout.

Willingness did not differ significantly according to previous probiotic awareness (Fisher’s exact *p* = 0.851). In the ordinal logistic regression model (Table 4), probiotic awareness, education, gender, age, yogurt-consumption frequency, and denture stomatitis were not independently associated with willingness to try a probiotic-based denture cleanser. The proportional-odds assumption was satisfied (Brant test, χ^2^ = 8.22, df = 10, *p* = 0.607). Confidence intervals were wide for sparsely populated categories, widest for postgraduate versus no formal education (OR 2.63; 95% CI 0.36–19.36), so the estimates are imprecise and no individual estimate should be read as informative. With 13 participants in the smallest response category and ten model parameters, this model was too small for stable estimation, so the absence of significant predictors should not be read as evidence of no association.

In terms of preferred delivery format, mouthwash was the most frequently selected option (34.6%), followed by a soaking tablet (18.7%), dietary form (13.1%), and gel (6.5%); 27.1% indicated that they were not interested in a specific probiotic-based cleanser format (Figure 3b). Because one response option (dietary form) describes a patient-delivered product rather than a cleanser, the format item was broader in scope than the willingness item.

### 3.8. Exploratory Associations Between Probiotic Awareness and Clinical Outcomes

In exploratory analyses, denture stomatitis, worst-arch DCI, and worst-arch biofilm accumulation did not differ significantly between participants who were aware of oral probiotics and those who were not (*p* = 0.294, *p* = 0.277 and *p* = 0.130, respectively; Table 5). Given the small aware subgroup (n = 20), these analyses had limited power, and no inference about an association can be drawn.

## 4. Discussion

This cross-sectional study included 107 wearers of removable prostheses attending the Prosthodontic Clinics at the College of Dentistry, King Saud University. A clear gap was found between the perceived importance of denture hygiene and the reported use of evidence-based cleaning methods. Awareness of the oral microbiome and oral probiotics was low, although willingness to try a probiotic-based denture cleanser was common. Neither the composite knowledge/awareness score nor self-reported cleaning frequency was associated with objective denture cleanliness or biofilm accumulation. Denture stability differed among prosthesis types in the omnibus test only. Female participants had lower adjusted odds of both a low knowledge score and poor denture cleanliness, and no sociodemographic or clinical factor independently predicted stated willingness.

### 4.1. The Knowledge–Practice Gap

Almost all participants (97.2%) considered denture cleaning important. However, fewer than one-quarter used a denture-cleansing tablet. Toothpaste and water alone were the most frequently reported methods, despite established concerns that toothpaste may abrade denture materials [35], whereas water alone is less effective than chemical denture cleansers at reducing microbial adherence [36]. This dissociation between attitude and practice mirrors findings from other Saudi and regional samples. In the Eastern Province of Saudi Arabia, most complete-denture wearers reported daily cleaning, but few used cleansing tablets and many used toothpastes [12]. Similar gaps have been reported among removable partial denture wearers in the United Kingdom [11] and in other cross-sectional studies of denture hygiene [10].

The present study adds an objective clinical assessment to these observations. Neither cleaning frequency nor the composite knowledge score was correlated with DCI or biofilm accumulation. This does not mean that cleaning practices are unimportant. Objective cleanliness also depends on cleaning technique, product choice, contact time with the cleanser, prosthesis surface characteristics and manual dexterity, none of which were measured here. Reported practices are themselves subject to social desirability bias, which would further weaken any association with clinical findings. Older participants had higher biofilm scores (Section 3.6), so age-related dexterity may be one contributor. Educational interventions should therefore provide practical guidance on appropriate products and cleaning techniques rather than focusing only on cleaning frequency.

In the present sample, verbal instruction from a dentist was the main source of denture-care information for 83.2% of participants, and 10.3% had never received any instruction, so education was both narrow in format and incomplete in reach. A similar pattern has been reported elsewhere in Saudi Arabia, where 93.3% of patients who had received post-insertion instructions received them orally [15]. Format appears to matter: in a comparative trial of patient-education formats, denture plaque scores did not improve significantly over one week among patients instructed verbally, whereas audio and video instruction produced significantly lower plaque scores [37]. Practitioner preparation may be a further constraint, as many practitioners have reported inadequate undergraduate training in recommending denture cleansers [14]. No Saudi data describe clinicians’ knowledge of denture cleansers, which remains an obvious target for future work.

### 4.2. Low Probiotic Awareness and Common Stated Willingness

Awareness of the oral microbiome (17.8%) and oral probiotics (18.7%) was low. Nevertheless, 51.4% of participants said they would try a probiotic-based denture cleanser and a further 35.5% were undecided. None of the examined sociodemographic or clinical factors was significantly associated with willingness in the multivariable model, although the model was too small to exclude modest associations. These findings indicate broad interest in probiotic-based denture cleansers within this sample. However, reported willingness does not necessarily predict product acceptance or sustained use. Responses to this item were also unstable on retest (Section 3.1), which supports reading them as a provisional reaction rather than a settled intention. Further studies should assess acceptability, feasibility, and adherence.

Trials in denture wearers have reported that probiotic strains such as *Lactobacillus rhamnosus* and *Streptococcus salivarius* K12 can reduce *Candida* colonization and the symptoms of denture stomatitis [20,21,22,38]. This evidence is heterogeneous. Studies differ in strain, dose, vehicle, treatment duration and outcome measures, and most are small and short-term [19,39]. Laboratory work suggests that oral *lactobacilli* and related species can modulate biofilm composition and inhibit pathogenic colonization [17,18]. In all of these studies, however, the probiotic was taken by the patient rather than applied to the prosthesis, so none of them tests a probiotic-based denture cleanser. Mouthwash was the most frequently preferred format. The reasons for this preference were not examined. Acceptance of an actual product would also depend on ease of use, taste, cost, adherence over time and compatibility with denture base materials, none of which were assessed. Format preference is therefore a starting point for product design, not evidence of acceptability.

### 4.3. Clinical Findings by Prosthesis Type

Denture stability differed among prosthesis types in the omnibus test, but no pairwise comparison remained significant after adjustment for multiple testing. The RPD-M subgroup was also small, and stability data were missing for one participant. These data therefore do not establish that any prosthesis type provides better stability. Retention, fit, DCI and biofilm accumulation did not differ among prosthesis types, and the mandibular-arch analysis showed the same pattern (Section 3.5).

Denture stomatitis was present in 14.2% of clinically examined participants. Mild stomatitis was observed in 11.3%, and moderate stomatitis in 2.8%; no severe cases were recorded. This prevalence was lower than the 23.4% reported in another Saudi population [8]. Differences in sampling, clinical setting, examiner calibration, or regional characteristics may explain this variation. Comparisons among prevalence studies are also limited by differences in diagnostic criteria and examiner training [3,33].

### 4.4. Gender Differences

Female gender was associated with lower odds of both a low knowledge score and poor denture cleanliness. The direction is consistent with evidence from Jeddah, where women scored higher on oral health knowledge and attended more often for prevention than men [40]. It also agrees with international reviews of sex differences in oral hygiene behavior and oral health literacy [41]. The association was weaker when the knowledge score was analyzed as a continuous outcome (Section 3.4), so it should be read as provisional rather than established. The size of the association with poor cleanliness also varied with the arch definition (OR 0.18–0.29). Complete-denture wearers in the Eastern Province showed the opposite pattern for patient-reported outcomes, with women reporting worse oral health impact scores than men [12]. Oral health-related quality of life was not assessed here. Whether better hygiene among women coexists with a less favorable subjective denture experience therefore cannot be determined, and this warrants dedicated study combining objective and patient-reported measures.

### 4.5. Strengths and Limitations

A major strength of this study was the pairing of a questionnaire assessed for content validity and test–retest reliability with a standardized clinical examination. A single calibrated examiner performed all clinical assessments. This design allowed direct comparison of self-reported practices with objective denture hygiene outcomes. A structured literature search identified no previous Saudi study reporting denture wearers’ awareness of oral probiotics or their willingness to use a probiotic-based denture cleanser, although work published in sources not indexed in the databases searched cannot be excluded.

Several limitations should be considered. The cross-sectional design prevents causal inference. The sample size was calculated to estimate the primary prevalence-based objectives and was not independently powered for the secondary group comparisons, association analyses, and multivariable models reported. These findings should be interpreted as exploratory and hypothesis-generating. Convenience sampling from one academic dental hospital may also limit generalizability to the wider Saudi denture-wearing population.

The use of a single calibrated examiner reduced inter-examiner variation but did not permit assessment of inter-examiner agreement. Systematic measurement bias cannot be excluded either. Self-reported practices may have been affected by recall and social desirability bias. Some prosthesis subgroups were small, particularly the RPD-M group, which limited the power and precision of comparisons. The multivariable models for poor denture cleanliness and for willingness were fitted with fewer than ten events per variable, so they are at risk of overfitting and their estimates are unstable. The size of the gender association with poor cleanliness varied with the arch definition, and the null findings of the willingness model should not be read as evidence of no association. Both models should be regarded as hypothesis-generating.

Awareness and knowledge of the oral microbiome and probiotics were assessed by self-report rather than validated microbiological testing. Clinical signs suggestive of fungal infection were not confirmed microbiologically; therefore, no conclusions can be drawn about *Candida* carriage. Microbiological analyses of samples from this cohort, including quantitative PCR for selected bacterial and fungal targets, are in preparation and will be reported separately. Finally, because prosthesis type frequently differed between the upper and lower arch within the same participant, the prosthesis-type comparisons in this study were restricted to the upper arch as the representative unit of analysis. This approach preserves statistical independence but means findings may not generalize directly to mandibular prostheses, where retention and stability are known to differ systematically from maxillary prostheses. Halitosis was recorded as a dichotomous clinical judgment without a standardized organoleptic protocol or instrumental measurement, and was not separately validated in this sample. Apart from age, the measured correlates of cleanliness (duration and frequency of denture wear, cleaning method and adhesive use) showed no association with objective findings, so the dissociation more likely reflects unmeasured factors such as cleaning technique and manual dexterity.

The questionnaire also has limitations. It was purpose-designed and assessed only for content validity and test–retest reliability; its construct validity and internal consistency were not examined. Seven items fell below the I-CVI threshold on at least one dimension and were retained after their wording was simplified, without re-rating by the panel (Section 3.1). The reliability subsamples were small (14 participants for the questionnaire and 8 for the clinical examination), so the agreement estimates are imprecise. The willingness item showed only slight test–retest agreement (κw = 0.169), so the willingness findings are preliminary. The four items of the composite knowledge score were weighted equally without an empirical basis, and the score is a descriptive index rather than a validated scale. Finally, the Arabic version underwent forward translation, expert review and pilot testing but not independent back-translation, so subtle differences in meaning between the two language versions cannot be excluded.

### 4.6. Implications and Future Directions

These findings suggest two priorities for future work. First, perceived importance of denture hygiene was high while the use of evidence-based cleaning methods was low. Education should therefore address practical technique and product choice rather than general awareness, and should include dental practitioners, whose training in denture cleansers has been reported as inadequate [14]. Second, probiotic awareness was low, but stated willingness to try such a product was common. No significant predictors of willingness were identified, although this may partly reflect the sample size. These findings alone do not establish the clinical value of probiotic-based denture cleansers. However, when considered alongside emerging laboratory and clinical evidence, they support further evaluation in Saudi denture wearers [17,18,19,20,21,22,38,39]. Initial studies should assess formulation acceptability, safety, feasibility, and adherence. If these findings are favorable, controlled clinical trials could evaluate probiotic-based denture cleansers as an adjunct to established denture hygiene measures.

## 5. Conclusions

Among removable-prosthesis wearers attending a Saudi university dental hospital, denture hygiene was widely regarded as important, yet evidence-based cleaning methods were used infrequently. Neither self-reported knowledge nor cleaning frequency corresponded to objectively measured denture cleanliness. Awareness of the oral microbiome and of oral probiotics was low. Half of the participants said they would try a probiotic-based denture cleanser and a further third were undecided, which indicates openness to the concept rather than demonstrated acceptance. Because responses to this item were unstable on retest, this finding is preliminary. These findings support evidence-based denture-hygiene education for patients and practitioners, and controlled clinical trials to determine whether probiotic-based denture cleansers are effective and safe adjuncts to conventional hygiene.

## Figures and Tables

**Figure 1:**
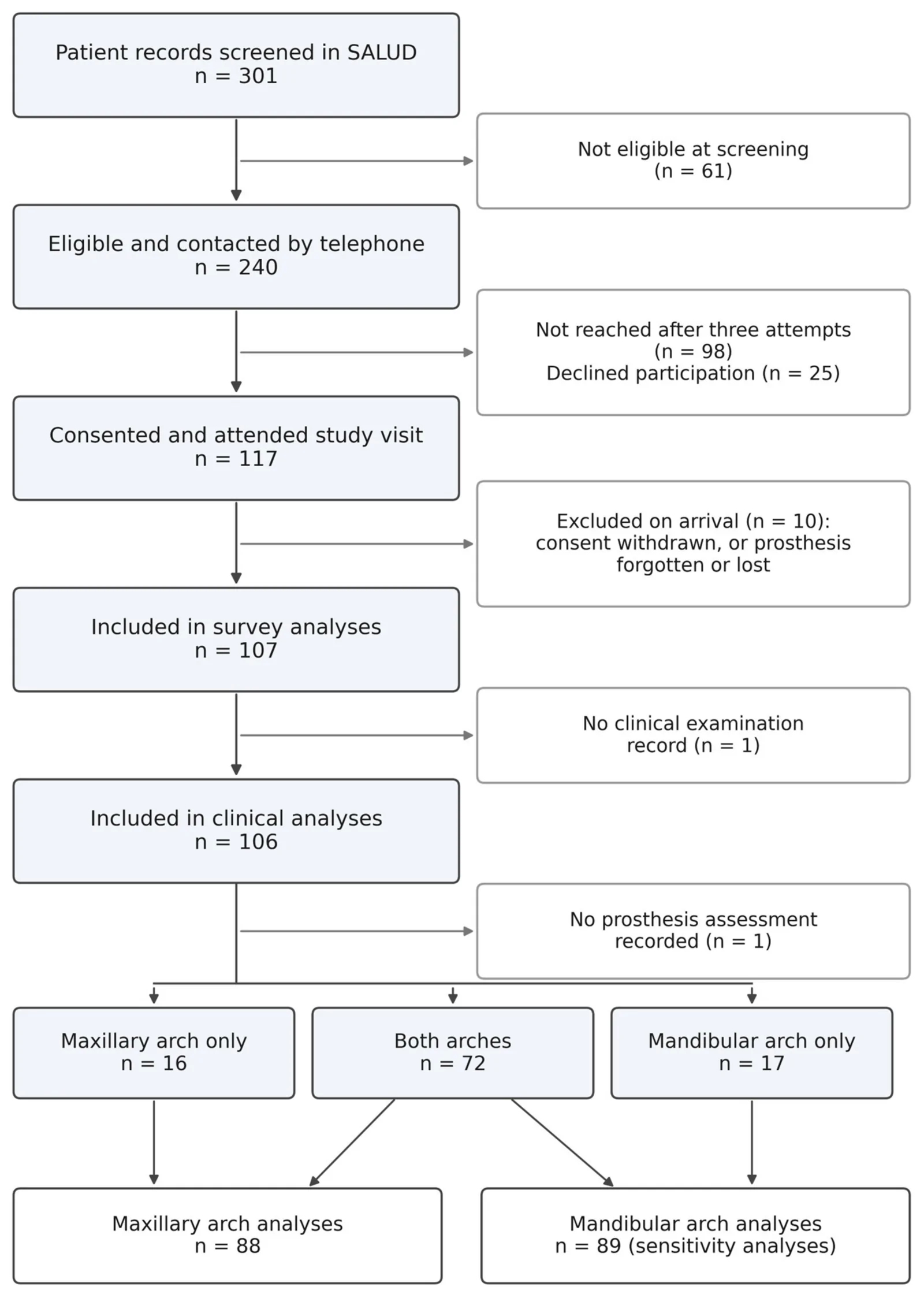
Participant recruitment and inclusion flow, with the number of participants contributing to the maxillary and mandibular analyses.

**Figure 2:**
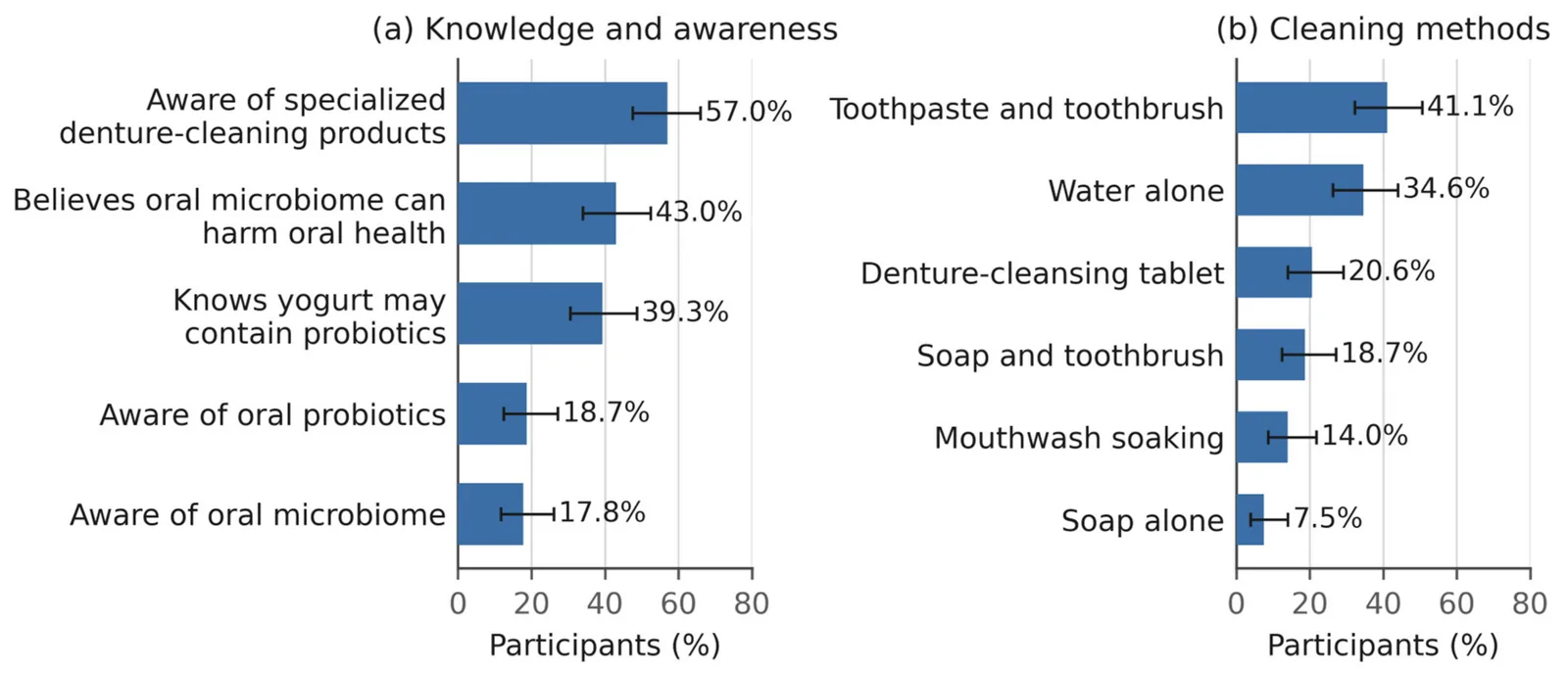
Knowledge and awareness items (**a**) and practice items, namely reported denture-cleaning methods (**b**), expressed as the percentage of participants (N = 107). Cleaning methods were not mutually exclusive, so percentages sum to more than 100%. Error bars show 95% confidence intervals (Wilson method).

**Figure 3:**
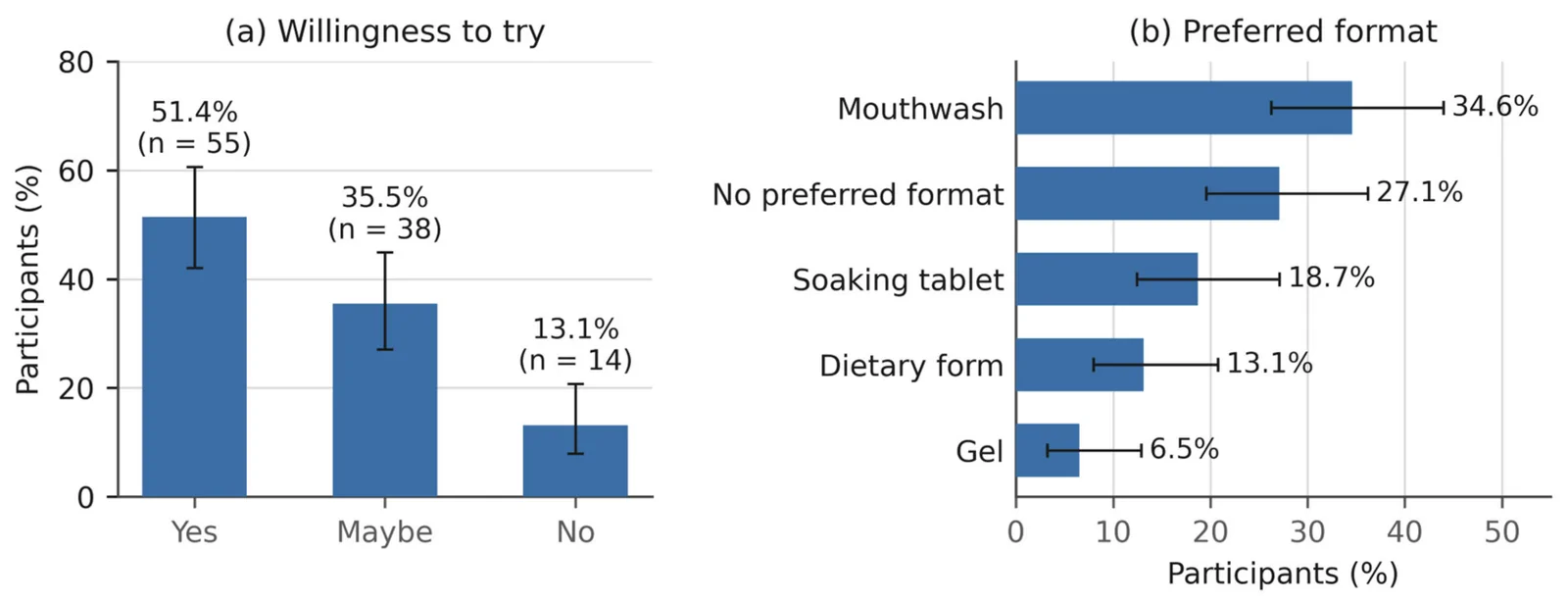
Stated willingness to try a probiotic-based denture cleanser (**a**) and preferred product format (**b**) (N = 107). Error bars show 95% confidence intervals (Wilson method).

**Table 1:** Sociodemographic, Medical, and Denture-History Characteristics of the Study Sample.

Section	Characteristic	Findings, n (%) Unless Noted (N = 107)
Sociodemographic Characteristics	Age (years)	Mean (SD) 58.6 (13.2); Median (IQR) 60 (50–68)
	Age group	21–40 y: 8 (7.5%); 41–60 y: 49 (45.8%); 61–80 y: 48 (44.9%); >80 y: 2 (1.9%)
	Gender	Male: 44 (41.1%); Female: 63 (58.9%)
	Nationality	Saudi: 78 (72.9%); non-Saudi: 29 (27.1%)
	Marital status	Married: 81 (75.7%); Divorced/Separated: 6 (5.6%); Widowed: 10 (9.3%); Single/Never married: 10 (9.3%)
	Education level	Illiterate/No formal: 17 (16.0%); Primary: 17 (16.0%); Secondary: 33 (31.1%); University: 32 (30.2%); Postgraduate: 7 (6.6%); Unknown: 1
	Health-related background	Yes: 31 (29.0%)
	Employment status	Full-time: 38 (35.5%); Part-time: 30 (28.0%); Self-employed/Freelancer: 4 (3.7%); Student: 2 (1.9%); Retired/Unemployed: 33 (30.8%)
Medical History	Systemic medical condition	None: 36 (33.6%); Diabetic: 16 (15.0%); Hypertension: 6 (5.6%); Diabetes + Hypertension: 8 (7.5%); Combination of diabetes, hypertension and other: 26 (24.3%); Other: 15 (14.0%)
	Antibiotic use (past 3 months)	14 (13.1%)
	Respiratory infection (past 3 months)	18 (16.8%)
	Smoking status	Never-smoker: 92 (86.0%); Current smoker: 9 (8.4%); Ex-smoker: 6 (5.6%)
Denture History	Duration of current denture use	<1 y: 55 (51.4%); 1–2 y: 16 (15.0%); 3–4 y: 12 (11.2%); 4–5 y: 6 (5.6%); >5 y: 18 (16.8%)
	Frequency of dental attendance	Every <6 mo: 47 (43.9%); Every 6–12 mo: 19 (17.8%); Every 1–2 y: 6 (5.6%); As complaints arise: 31 (29.0%); Never: 4 (3.7%)
	Primary source of denture-care information	Verbal, from dentist: 89 (83.2%); Written, from dentist: 6 (5.6%); Non-dentist source: 1 (0.9%); Never received: 11 (10.3%)
	Frequency of denture wear	Continuously (incl. night): 31 (29.0%); Day only: 49 (45.8%); Occasionally: 27 (25.2%)
	Frequency of denture cleaning	After every meal: 64 (59.8%); Once daily (bedtime): 31 (29.0%); Once daily (morning): 4 (3.7%); Few times a week: 2 (1.9%); Rarely: 6 (5.6%)
	Denture adhesive use	Yes, regularly: 9 (8.4%); Occasionally: 15 (14.0%); No, never: 83 (77.6%)

Data are presented as n (%) unless otherwise indicated. Percentages are calculated based on non-missing responses for each variable; missing/unknown values are reported where applicable.

**Table 2:** Knowledge, Attitudes, and Practices Related to Oral Probiotics and the Oral Microbiome (N = 107).

Knowledge/Awareness Domain	Yes, n (%)	No, n (%)	Not Sure ^a^, n (%)
Perceived importance of denture cleaning	104 (97.2%)	2 (1.9%)	1 (0.9%)
Awareness of specialised cleaning products	61 (57.0%)	36 (33.6%)	10 (9.3%)
Awareness of oral microbiome	19 (17.8%)	70 (65.4%)	18 (16.8%)
Belief that the oral microbiome can negatively affect oral health	46 (43.0%)	24 (22.4%)	37 (34.6%)
Awareness of oral probiotics	20 (18.7%)	64 (59.8%)	23 (21.5%)
Knowledge that yogurt contains probiotics	42 (39.3%)	44 (41.1%)	21 (19.6%)
Previous use of a probiotic product	18 (16.8%)	72 (67.3%)	17 (15.9%)
Willingness to try a probiotic-based denture cleanser ^a^	55 (51.4%)	14 (13.1%)	38 (35.5%)
Composite knowledge score (range 0–4)	Mean (SD) 1.19 (1.05); Median (IQR) 1.00 (0.00–2.00)		

Data are presented as n (%) unless otherwise indicated. ^a^ Response options for this item were Yes/No/Maybe; “Maybe” responses are listed in the “Not sure” column. Composite knowledge score reflects correct responses across four probiotic/microbiome knowledge items (range 0–4); higher scores indicate greater knowledge.

**Table 3:** Clinical Denture and Oral Findings by Prosthesis Type in the Maxillary Arch, Used as the Single Representative Arch per Participant (n = 88).

Parameter	Category	CD (N = 50)	RPD-M (N = 14)	RPD-A (N = 24)	*p* Value
DCI score (0–4) ^a^	Median (IQR)	1.0 (0.0–2.0)	1.0 (0.0–2.0)	1.0 (0.0–2.0)	0.855
Biofilm accumulation (1–4) ^a^	Median (IQR)	2.0 (1.0–3.0)	2.0 (1.0–2.0)	1.5 (1.0–2.5)	0.536
Denture retention ^b^	No retention	5 (10.0%)	1 (7.7%)	7 (29.2%)	0.090
	Minimal	12 (24.0%)	0 (0.0%)	3 (12.5%)	
	Moderate	15 (30.0%)	3 (23.1%)	6 (25.0%)	
	Good	18 (36.0%)	9 (69.2%)	8 (33.3%)	
Denture stability ^b^	No stability	6 (12.0%)	1 (7.7%)	7 (29.2%)	0.038 *
	Some	21 (42.0%)	2 (15.4%)	10 (41.7%)	
	Sufficient	23 (46.0%)	9 (69.2%)	7 (29.2%)	
	Good	0 (0.0%)	1 (7.7%)	0 (0.0%)	
Denture fit ^b^	Ill-fitting/fractured	9 (18.0%)	2 (15.4%)	7 (29.2%)	0.300
	Minor misfit	19 (38.0%)	2 (15.4%)	8 (33.3%)	
	Well-fit	22 (44.0%)	9 (69.2%)	9 (37.5%)	
Denture stomatitis ^c^ (any Newton type)	Present	9 (18.0%)	1 (7.1%)	5 (20.8%)	0.600

CD = complete denture; RPD-A = acrylic removable partial denture; RPD-M = metal-framework removable partial denture; DCI = Denture Cleanliness Index. * *p* < 0.05. ^a^ Kruskal–Wallis test. ^b^ Fisher’s exact test (Monte Carlo simulation, 10,000 resamples). Higher categories indicate better denture quality. RPD-M *n* = 13 (one participant with missing data). ^c^ Fisher’s exact test (Monte Carlo simulation, 10,000 resamples). Presence of any Newton type (Types I and II were observed), assessed at the whole-mouth level (RPD-M *n* = 14). The full distribution is given in Section 3.5.

**Table 4:** Multivariable Analyses of Predictors of a Lower Knowledge Score, Poor Denture Hygiene, and Stated Willingness to Try a Probiotic-Based Denture Cleanser.

Outcome (Model)	Predictor	Adjusted OR (95% CI)	*p* Value
Predictors of a Lower Knowledge Score (composite score 0–1 vs. 2–4) Binary logistic regression n = 106; EPV = 10.0 (40 events, 4 predictors)	Age (years)	0.972 (0.934–1.008)	0.147
	Gender: Female vs. Male	0.290 (0.101–0.764)	0.016 *
	Education level	0.777 (0.518–1.144)	0.208
	Nationality: Non-Saudi vs. Saudi	0.576 (0.227–1.451)	0.240
Predictors of Poor Denture Cleanliness Index (worst-arch DCI ≥ 3) Binary logistic regression n = 103; EPV = 5.0 (25 events, 5 predictors)	Denture cleaning frequency	1.050 (0.562–1.777)	0.863
	Knowledge score	1.431 (0.893–2.351)	0.142
	Gender: Female vs. Male	0.187 (0.055–0.566)	0.004 *
	Age (years)	1.010 (0.971–1.052)	0.619
	Education level	0.841 (0.527–1.326)	0.455
Predictors of Stated Willingness to Use a Probiotic-Based Denture Cleanser (No < Maybe < Yes) Ordinal logistic regression (proportional odds) n = 106; EPV = 1.3 (13 in smallest category, 10 parameters)	Probiotic awareness: No vs. Yes	1.123 (0.391–3.219)	0.830
	Probiotic awareness: Not sure vs. Yes	1.409 (0.417–4.767)	0.581
	Education: Primary vs. Illiterate	1.085 (0.279–4.214)	0.906
	Education: Secondary vs. Illiterate	0.859 (0.263–2.806)	0.802
	Education: University vs. Illiterate	0.751 (0.211–2.674)	0.658
	Education: Postgraduate vs. Illiterate	2.630 (0.357–19.357)	0.342
	Gender: Female vs. Male	1.180 (0.492–2.831)	0.711
	Age (years)	0.992 (0.957–1.027)	0.644
	Yogurt intake frequency	0.952 (0.671–1.351)	0.782
	Stomatitis present	0.910 (0.300–2.759)	0.867

OR = adjusted odds ratio; CI = confidence interval; EPV = events per variable (for the ordinal model, participants in the smallest response category per model parameter); values below 10 indicate a risk of overfitting. * *p* < 0.05. Lower knowledge score model: outcome coded 0–1 vs. 2–4, corresponding to the sample median (see Methods); OR < 1 indicates lower odds of a score of 0–1. Poor DCI model: outcome coded worst-arch Denture Cleanliness Index ≥ 3 (poor) vs. ≤2 (acceptable), per the published DCI classification [31]; OR < 1 indicates lower odds of poor hygiene. Willingness model: ordinal outcome (No < Maybe < Yes) analyzed by proportional-odds logistic regression; OR > 1 indicates higher odds of being in a higher willingness category. Education was modeled as an ordinal predictor in the lower-knowledge-score and poor-DCI models, and as a categorical predictor (reference: Illiterate/No formal education) in the willingness model; education was not a significant predictor of any outcome regardless of coding. The poor-cleanliness model was fitted in 103 participants with complete data (3 participants had no recorded Denture Cleanliness Index and 1 had missing education data); 25 met the outcome definition. Confidence intervals are profile-likelihood intervals for the two binary logistic models and Wald intervals for the ordinal model.

**Table 5:** Exploratory Associations Between Prior Probiotic Awareness and Clinical Outcomes.

Outcome	Aware (n = 20)	Not Aware/Uncertain (n = 87)	Test	*p* Value
Stomatitis present, n (%)	1 (5.0%)	14 (16.3%)	Fisher’s exact (simulated)	0.294
Worst-arch DCI, median (IQR)	1 (0–2)	1 (0–2.25)	Kruskal–Wallis H(1) = 1.182	0.277
Worst-arch biofilm, median (IQR)	1 (1–3)	2 (1–3)	Kruskal–Wallis H(1) = 2.295	0.130

Fisher’s exact test used Monte Carlo simulation (10,000 resamples). Probiotic-aware = responded ‘Yes’ to prior awareness of oral probiotics. Not aware/uncertain = responded ‘No’ or ‘Not sure’. Group sizes refer to the survey sample. Clinical data were available for all 20 aware participants, and for 86 (stomatitis) and 84 (DCI and biofilm) not aware/uncertain participants; percentages and medians are based on available data.

## Data Availability

The data presented in this study are available on reasonable request from the corresponding author. The data are not publicly available due to participant privacy and ethical restrictions.

## Supplementary Materials

The following supporting information can be downloaded at https://www.mdpi.com/article/10.3390/healthcare14203466/s1, File S1: Consent Form (Arabic and English versions); File S2: Study questionnaire (Arabic and English versions); File S3: Clinical examination form; File S4: Statistical analysis script (R) and diagnostics output; Table S1: Item-level content validity indices (I-CVI) for the study questionnaire, rated independently by four external expert consultants.

## Abbreviations

The following abbreviations are used in this manuscript:

CD	Complete denture
CI	Confidence interval
CVI	Content Validity Index
DCI	Denture Cleanliness Index
ICC	Intraclass correlation coefficient
IQR	Interquartile range
OR	Odds ratio
RPD-A	Acrylic removable partial denture
RPD-M	Metal-framework removable partial denture
SD	Standard deviation
