# Supplementary material for: Denture Hygiene Knowledge, Practices, and Stated Willingness to Use a Probiotic-Based Denture Cleanser Among Removable-Prosthesis Wearers at a Saudi University Dental Hospital: A Cross-Sectional Study
Source: Healthcare (Basel). 2026 Oct 15;14(20):3466. doi: 10.3390/healthcare14203466 (PMC13704227; doi:10.3390/healthcare14203466)
Supplement: Supplementary file 1 [file healthcare-14-03466-s001.zip › File S1 Consent Form (English and Arabic).pdf]

File no.	Contact no.	Date:

Oral Probiotics and Denture Care in Saudi Arabia: Evaluating Knowledge, Practices, and the Future of Innovative Hygiene Solutions

Principal investigator: Dr. Rua Babaier

Institution: King Saud University

You are invited to participate in a research study assessing oral and denture hygiene and identifying the types of microorganisms that colonize the mouths and dentures of adult denture wearers. This study also explores hygiene habits and health conditions that influence microbial presence.

If you agree to participate, you will:

1. Complete a short questionnaire (approx. 10 minutes) about your oral hygiene practices, denture use, and general health.
2. Undergo a clinical examination of your mouth and denture to evaluate hygiene and condition.

Duration: ~15–20 minutes

Participation: One-time only; no follow-up visits required.

Confidentiality:

- Your personal and medical information will remain confidential.
- Your name will not appear on any samples or reports.
- All data will be securely stored and used only for research purposes.

Voluntary Participation:

Participation is voluntary. You may decline or withdraw at any time without giving a reason. This will not affect your medical care.

☐ I have read (or had read to me) the information above. I understand the study's purpose, procedures, risks, and benefits. I had the chance to ask questions and received satisfactory answers.

**رقم الملف:**	**رقم الجوال:**	**التاريخ:**

المعززات الحيوية الفموية ونظافة الأطقم في السعودية: تقييم المعرفة والممارسات وآفاق حلول النظافة المبتكرة

الباحث الرئيس: د. رؤى بابعير;<RBabaier@KSU.EDU.SA >
الجهة: جامعة الملك سعود

ندعوك للمشاركة في دراسة بحثية تهدف إلى تقييم نظافة الفم والطقم وتحديد أنواع الميكروبات التي تستعمر فم وأطقم مستخدمي الأطقم من البالغين. كما تبحث الدراسة في العادات الصحية والحالة الطبية التي تؤثر على وجود هذه الميكروبات.

في حال موافقتك على المشاركة، ستقوم بـ:

- تعبئة استبيان قصير (حوالي 10 دقائق) عن ممارساتك في نظافة الفم، واستخدام الطقم، وصحتك العامة.
- الخضوع لفحص سريري للفم والطقم لتقييم حالتهما.

مدة المشاركة : من 15 إلى 20 دقيقة تقريبًا
عدد المشاركات : مرة واحدة فقط، ولا توجد زيارات متابعة.

السرية:

- ستبقى معلوماتك الشخصية والطبية سرية تمامًا.
- لن يظهر اسمك في أي عينة أو تقرير منشور.
- سيتم تخزين البيانات بأمان واستخدامها لأغراض البحث فقط.

المشاركة طوعية تمامًا:
يحق لك رفض المشاركة أو الانسحاب في أي وقت دون إبداء السبب، ولن يؤثر ذلك على رعايتك الصحية بأي شكل.

☐ لقد قرأت (أو تمت قراءته لي) المعلومات الواردة أعلاه. وفهمت الهدف من الدراسة، وإجراءاتها، ومخاطرها، وفوائدها. وأُتيحت لي الفرصة لطرح الأسئلة، وتمت الإجابة عليها بشكل مرضٍ.
