# Supplementary material for: Denture Hygiene Knowledge, Practices, and Stated Willingness to Use a Probiotic-Based Denture Cleanser Among Removable-Prosthesis Wearers at a Saudi University Dental Hospital: A Cross-Sectional Study
Source: Healthcare (Basel). 2026 Oct 15;14(20):3466. doi: 10.3390/healthcare14203466 (PMC13704227; doi:10.3390/healthcare14203466)
Supplement: Supplementary file 1 [file healthcare-14-03466-s001.zip › File S2 questionnaire (Survey-P1, Arabic + English).pdf]

PQ#...

File no.	Contact no.	Date:

Oral Probiotics and Denture Care in Saudi Arabia: Evaluating Knowledge, Practices, and the Future of Innovative Hygiene Solutions

Principal investigator: Dr. Rua Babaier

Institution: King Saud University

You are invited to participate in a research study assessing oral and denture hygiene and identifying the types of microorganisms that colonize the mouths and dentures of adult denture wearers. This study also explores hygiene habits and health conditions that influence microbial presence.

If you agree to participate, you will:

1. Complete a short questionnaire (approx. 10 minutes) about your oral hygiene practices, denture use, and general health.
2. Undergo a clinical examination of your mouth and denture to evaluate hygiene and condition.

Duration: ~15–20 minutes

Participation: One-time only; no follow-up visits required.

Confidentiality:

- Your personal and medical information will remain confidential.
- Your name will not appear on any samples or reports.
- All data will be securely stored and used only for research purposes.

Voluntary Participation:

Participation is voluntary. You may decline or withdraw at any time without giving a reason. This will not affect your medical care.

☐ I have read (or had read to me) the information above. I understand the study's purpose, procedures, risks, and benefits. I had the chance to ask questions and received satisfactory answers.

PQ#…

Patient Survey (5 sections)

V.03/2025

**Section 1: Demographics and General Information**		
**1**	**Age:**	-----
**2**	**Gender:**	☐ Female ☐ Male
**3**	**Nationality:**	☐ Saudi ☐ Non-Saudi
**4**	**Marital status:**	☐ Married ☐ Divorced ☐ Widowed ☐ Single, never married
	***If you have children, what is the age of your youngest?**	------------
**5**	**Educational Level:**	☐ No formal education ☐ Primary<br>☐ Secondary ☐ University ☐ Postgraduate
	**Do you have a health-related background?**	☐ Yes ☐ No
**6**	**Employment Status:**	☐ Unemployed ☐ Employed full-time ☐ Employed part-time ☐ Freelancer ☐ Retired
**7**	**Do you have any medical conditions?**	☐ No ☐ Yes, please specify…
	**Do you use any medication?**	☐ Diabetic meds ☐ Blood pressure meds ☐ Others Please specify ----
**8**	**Have you taken antibiotics in the last 3 months?**	☐ Yes ☐ No
**9**	**Have you experienced a respiratory infection or sore throat in the last 3 months?**	☐ Yes ☐ No
**10**	**Do you smoke?**	☐ None-smoker<br>☐ Current smoker (---packs/day)<br>☐ Previous smoker (---years quit smoking
**11**	**How long have you worn your current denture(s)?**	☐ Less than 1 year<br>☐ 1-2 years<br>☐ 3-4 years<br>☐ Less than 5 years<br>☐ more than 5 years
**12**	**How often do you visit the dentist for routine checkups?**	☐ 1 time in 6 months<br>☐ Once a year<br>☐ 1 time in 2 years<br>☐ As many complaints<br>☐ None

Section 2: Knowledge and Awareness		
**13**	**Where did you learn about denture care?**	☐ Verbal instructions from a dentist<br>☐ Written instructions from a dentist<br>☐ A non-dentist source<br>☐ Never received information
**14**	**Do you believe cleaning dentures is essential?**	☐ Yes ☐ No ☐ Not sure
**15**<br><br>**16**	**Are you aware of specialized denture cleaning products (tablets, mouthwash, and brushing)?**<br>**Have you heard of the oral microbiome?**	☐ Yes ☐ No ☐ Not sure<br><br>☐ Yes ☐ No ☐ Not sure
**17**	**Do you think that all oral microbiomes are harmful?**	☐ Yes ☐ No ☐ Not sure
**18**	**Have you ever heard of oral probiotics (beneficial bacteria)?**	☐ Yes ☐ No ☐ Not sure
**19**	**Do you know that yoghurt contains probiotics (beneficial bacteria)?**	☐ Yes ☐ No ☐ Not sure

Section 3: Current Denture Hygiene Practices		
**20**	**How frequently do you wear your current denture?**	☐ All the time ☐ Remove them at night<br>☐ Meals / social activities
**21**	**How often do you clean your dentures?**	☐ After every meal ☐ Once daily before bedtime ☐ Once daily in morning ☐ A few times a week ☐ Rarely
**22**	**Do you use denture adhesives?**	☐ Yes, always ☐ Sometimes ☐ No, never
**23**	**How do you clean your dentures?** (Multiple selection possible)	☐ I don't clean them<br>☐ Water only<br>☐ Soap only<br>☐ Bleach<br>☐ Soap and toothbrush<br>☐ Toothpaste and toothbrush<br>☐ Baking soda and a toothbrush<br>☐ Soaking the denture in a cleaning tablet<br>☐ Soaking the denture in mouthwash<br>☐ Other ____________
**24**	**Have you used oral probiotics (beneficial bacteria) as mouthwash, lozenges, or supplements?**	☐ Yes ☐ No ☐ Not sure
**25**	**How often do you eat yoghurt/ drink Laban?**	☐ Daily ☐ 2-3 times a week<br>☐ once a week ☐ Rarely ☐ Never
	**How often do you eat probiotic-containing yoghurts such as Activia and Kefir?**	☐ Daily ☐ 2-3 times a week<br>☐ once a week ☐ Rarely ☐ Never

Section 4: Denture-Related Problems		
**26**	**Have you experienced oral fungal infections (e.g., thrush, denture stomatitis) since wearing your current dentures?**	☐ Yes ☐ No ☐ Not sure
**27**	**Have you experienced bad breath since getting your current dentures?**	☐ Yes, often ☐ Sometimes ☐ No
**28**	**Have you experienced discomfort, redness, or irritation under your current dentures?**	☐ Yes, often ☐ Sometimes ☐ No

Section 5: Perceived Need for a New Denture Cleanser		
**29**	**Would you like to try a probiotic-based denture cleanser?**	☐ Yes ☐ No ☐ Maybe
**30**	**What form of denture cleanser would you prefer?**	☐ Dietary form<br>☐ Soaking tablet<br>☐ Mouthwash<br>☐ Gel<br>☐ Not interested

We sincerely appreciate your time and contribution in completing this questionnaire.

Your participation is a valuable contribution to scientific research and will help improve future oral healthcare for denture wearers.

With sincere appreciation,

The Research Team

PQ#...

**رقم الملف:**	رقم الجوال:	التاريخ:

المعززات الحيوية الفموية ونظافة الأطقم في السعودية: تقييم المعرفة والممارسات وآفاق حلول النظافة المبتكرة

الباحث الرئيس: د. رؤى بابعير;<RBabaier@KSU.EDU.SA >
الجهة: جامعة الملك سعود

ندعوك للمشاركة في دراسة بحثية تهدف إلى تقييم نظافة الفم والطقم وتحديد أنواع الميكروبات التي تستعمر فم وأطقم مستخدمي الأطقم من البالغين. كما تبحث الدراسة في العادات الصحية والحالة الطبية التي تؤثر على وجود هذه الميكروبات.

في حال موافقتك على المشاركة، ستقوم بـ:

- تعبئة استبيان قصير (حوالي 10 دقائق) عن ممارساتك في نظافة الفم، واستخدام الطقم، وصحتك العامة.
- الخضوع لفحص سريري للفم والطقم لتقييم حالتهما.

مدة المشاركة : من 15 إلى 20 دقيقة تقريبًا
عدد المشاركات : مرة واحدة فقط، ولا توجد زيارات متابعة.

السرية:

- ستبقى معلوماتك الشخصية والطبية سرية تمامًا.
- لن يظهر اسمك في أي عينة أو تقرير منشور.
- سيتم تخزين البيانات بأمان واستخدامها لأغراض البحث فقط.

المشاركة طوعية تمامًا:
يحق لك رفض المشاركة أو الانسحاب في أي وقت دون إبداء السبب، ولن يؤثر ذلك على رعايتك الصحية بأي شكل.

☐ لقد قرأت (أو تمت قراءته لي) المعلومات الواردة أعلاه. وفهمت الهدف من الدراسة، وإجراءاتها، ومخاطرها، وفوائدها. وأُتيحت لي الفرصة لطرح الأسئلة، وتمت الإجابة عليها بشكل مرضٍ.

PQ#...

استبيان المريض

V.02/2025 (5 أقسام)

الاستبيان

**القسم الأول: المعلومات الديموغرافية والعامة**		
**1**	**العمر:**	----
**2**	**الجنس:**	☐ أنثى ☐ ذكر
**3**	**الجنسية:**	☐ سعودي/ة ☐ غير سعودي/ة
**4**	**الحالة الاجتماعية:**	☐ متزوج ☐ مطلق ☐ أرمل ☐ أعزب
	إذا كان لديك أطفال، ما هو عمر أصغر طفل؟	**----**
**5**	**المستوى التعليمي:**	☐ بدون تعليم ☐ ابتدائي ☐ ثانوي ☐ جامعي ☐ دراسات عليا
	هل لديك خلفية في القطاع الصحي؟	☐ نعم ☐ لا
**6**	**الوضع الوظيفي:**	☐ غير موظف ☐ موظف بدوام كامل ☐ موظف بدوام جزئي ☐ مستقل ☐ متقاعد
**7**	**هل لديك حالات طبية؟**	☐ لا ☐ نعم (يرجى التحديد): ----
	**هل تستخدم علاج لها؟**	☐ علاج للسكر ☐ علاج لضغط الدم ☐ أدوية أخرى يرجى ذكرها ----
**8**	**هل استخدمت مضادات حيوية خلال الأشهر الثلاثة الماضية؟**	☐ نعم ☐ لا
**9**	**هل أصبت بعدوى في الجهاز التنفسي العلوي أو التهاب بالحلق خلال الثلاثة أشهر الماضية؟**	☐ نعم ☐ لا
**10**	**هل تدخن؟**	☐ لا ☐ نعم، (--- في اليوم) ☐ مدخن سابق منذ --- سنة
**11**	**منذ متى تستخدم طقم الأسنان (التركيبة السنية المتحركة) الحالي؟**	☐ أقل من سنة ☐ من سنة إلى سنتين ☐ من 3 إلى 4 سنوات ☐ 5 سنوات ☐ أكثر من 5 سنوات
**12**	**كم مرة تزور طبيب الأسنان للفحص الدوري؟**	☐ كل 6 أشهر ☐ سنويًا ☐ كل سنتين ☐ فقط عند وجود مشكلة ☐ لا أزور أبدًا

القسم الثاني: المعرفة والوعي		
13	من أين حصلت على معلومات حول العناية بطقم الأسنان الخاص بك ؟	☐ تعليمات شفوية من طبيب الأسنان<br>☐ تعليمات مكتوبة من طبيب الأسنان<br>☐ مصدر غير طبيب<br>☐ لم أستلم أي معلومات
14	هل تعتقد أن تنظيف طقم الأسنان الخاص بك أمر ضروري؟	☐ نعم ☐ لا ☐ غير متأكد
15	هل تعرف بوجود منتجات متخصصة لتنظيف أطقم الأسنان (مثل الحبوب الفوارة، غسول، الفرشاة)؟	☐ نعم ☐ لا ☐ غير متأكد
16	هل سمعت عن الميكروبيوم الفموي؟	☐ نعم ☐ لا ☐ غير متأكد
17	هل تعتقد أن جميع الميكروبات الفموية ضارة؟	☐ نعم ☐ لا ☐ غير متأكد
18	هل سمعت عن البروبيوتيك الفموي (البكتيريا الحيوية)؟	☐ نعم ☐ لا ☐ غير متأكد
19	هل تعلم أن اللبن يحتوي على بروبيوتيك (البكتيريا الحيوية)؟	☐ نعم ☐ لا ☐ غير متأكد

القسم الثالث: ممارسات نظافة الطقم الحالية		
20	كم مرة ترتدي طقم الأسنان الحالي؟	☐ طوال الوقت ☐ أزيله قبل النوم ☐ فقط أثناء الوجبات أو الأنشطة الاجتماعية
21	كم مرة تنظف طقم الأسنان الخاص بك؟	☐ بعد كل وجبة ☐ مرة يوميًا قبل النوم ☐ مرة يوميًا عند الاستيقاظ ☐ عدة مرات أسبوعيًا ☐ نادرًا
22	هل تستخدم لاصق أو مثبت لطقم الأسنان الخاص بك؟	☐ دائمًا ☐ أحيانًا ☐ لا أبدًا
23	كيف تنظف طقم الأسنان الخاص بك؟ (اختر جميع الإجابات التي تنطبق)	☐ لا أنظفه<br>☐ ماء فقط ☐ صابون فقط ☐ مبيض<br>☐ صابون وفرشاة ☐ معجون وفرشاة<br>☐ بيكربونات الصوديوم وفرشاة<br>☐ نقع الطقم في قرص تنظيف<br>☐ نقع الطقم في غسول فم<br>☐ أخرى (حدد)___________ :
24	هل سبق أن استخدمت بروبيوتك فموي (البكتيريا الحيوية) مثل غسول، حبوب استحلاب، أو مكملات لتحسين صحة الفم؟	☐ نعم ☐ لا ☐ غير متأكد
25	كم مرة تتناول منتجات اللبن (للشرب أو زبادي)؟	☐ يوميًا ☐ 2–3 مرات أسبوعيًا ☐ مرة أسبوعيًا ☐ نادرًا<br>☐ أبدًا
	كم مرة تتناول منتجات اللبن التي تحتوي على بروبيوتك (البكتيريا الحيوية) مثل الكفير و أكتيفيا؟	☐ يوميًا ☐ 2–3 مرات أسبوعيًا ☐ مرة أسبوعيًا ☐ نادرًا<br>☐ أبدًا

**القسم الرابع: المشاكل المرتبطة بالطقم**		
**26**	**هل أصبت بعدوى فطرية فموية (مثل القلاع أو التهاب الفم المرتبط بالطقم) بعد استخدام طقم الأسنان الحالي؟**	☐ نعم ☐ لا ☐ غير متأكد
**27**	**هل تعاني من رائحة فم كريهة منذ استخدام طقم الأسنان الخاص بك الحالي؟**	☐ غالبًا ☐ أحيانًا ☐ لا
**28**	**هل شعرت بانزعاج، أو احمرار، أو تهيج تحت طقم الأسنان؟**	☐ غالبًا ☐ أحيانًا ☐ لا

**القسم الخامس: الآراء حول منتجات جديدة**		
**29**	**هل ترغب في تجربة منظف أطقم أسنان يحتوي على بروبيوتك (البكتيريا الحيوية)؟**	☐ نعم ☐ لا ☐ ربما
**30**	**ما الشكل الذي تفضله لمنظف أطقم الأسنان؟**	☐ صالح للأكل ☐ قرص فوّار ☐ غسول فم ☐ جل<br>☐ غير مهتم

شكرًا لمشاركتك في هذا البحث
نشكر لك وقتك ومساهمتك في تعبئة هذا الاستبيان.

إن مشاركتك تُمثل مساهمة قيّمة في دعم البحث العلمي وتحسين مستوى الرعاية الصحية المقدمة للمستخدمين لأطقم الأسنان في المستقبل.

مع خالص التقدير،
