# Supplementary material for: Denture Hygiene Knowledge, Practices, and Stated Willingness to Use a Probiotic-Based Denture Cleanser Among Removable-Prosthesis Wearers at a Saudi University Dental Hospital: A Cross-Sectional Study
Source: Healthcare (Basel). 2026 Oct 15;14(20):3466. doi: 10.3390/healthcare14203466 (PMC13704227; doi:10.3390/healthcare14203466)
Supplement: Supplementary file 1 [file healthcare-14-03466-s001.zip › File S3 clinical examination form.pdf]

P no.	File no.	Contact no.	Date:

Clinical Examination Form

Section 1: Denture Assessment (Upper Jaw)		
****Is the patient wearing an upper denture?**		☐ Yes ☐ No
**1**	**Classification of the prosthesis:**	☐ Kennedy class I<br>☐ Kennedy class II<br>☐ Kennedy class III<br>☐ Kennedy class IV<br>☐ Complete edentulous
**2**	**Removable Denture Type:**	☐ Conventional<br>☐ Temporary/ Transitional/ Immediate<br>☐ Overdenture (teeth/implants)
**3**	**Prosthesis Material:**	☐ Polymethyl methacrylate<br>☐ Metallic framework (cobalt chromium)
**4**	**Abutments, if any:**	☐ None ☐ Teeth ☐ Implants ☐ Both
**5*****	**Condition of the remaining teeth, if any:**	☐ None ☐ Sound ☐ Fillings ☐ Crowns and Bridges ☐ defected/caries
**6**	**Denture Cleanliness Index (DCI) based on staining on the fitting surface:**	☐ 0 (Excellent) ☐ 1 ☐ 2 ☐ 3 ☐ 4 (Poor)
**7**	**Biofilm Accumulation on Denture:**	☐ None ☐ Mild ☐ Moderate ☐ Heavy
**8**	**Prosthesis retention:**	☐ No retention<br>☐ Minimum retention<br>☐ Moderate retention<br>☐ Good retention
**9**	**Prosthesis Stability:**	☐ No stability<br>☐ Some stability<br>☐ Sufficient stability
**10**	**Prosthesis fitting:**	☐ Well-fitting<br>☐ With minor misfit<br>☐ Completely ill-fitting or fractured
**11**	**Upper Denture Images**	☐ Occlusal view ☐ Fitting view

Section 2: Denture Assessment (Lower Jaw)		
**Is the patient wearing a lower denture?**		☐ Yes ☐ No
**1**	**Classification of the prosthesis:**	☐ Kennedy class I<br>☐ Kennedy class II<br>☐ Kennedy class III<br>☐ Kennedy class IV<br>☐ Complete edentulous
**2**	**Removable Denture Type:**	☐ Conventional<br>☐ Temporary/ Transitional/ Immediate<br>☐ Overdenture (teeth/implants).
**3**	**Prosthesis Material:**	☐ Polymethyl methacrylate ☐ Metallic framework (cobalt chromium)
**4**	**Abutments, if any:**	☐ None ☐ Teeth ☐ Implants ☐ Both
**5***	**Condition of the remaining teeth, if any:**	☐ None ☐ Sound ☐ Fillings ☐ Crowns and Bridges ☐ Defected/caries
**6**	**Denture Cleanliness Index (DCI) based on staining on the fitting surface:**	☐ 0 (Excellent) ☐ 1 ☐ 2 ☐ 3 ☐ 4 (Poor)
**7**	**Biofilm Accumulation on Denture:**	☐ None ☐ Mild ☐ Moderate ☐ Heavy
**8**	**Prosthesis retention:**	☐ No retention<br>☐ Minimum retention<br>☐ Moderate retention<br>☐ Good retention
**9**	**Prosthesis Stability:**	☐ No stability<br>☐ Some stability<br>☐ Sufficient stability
**10**	**Prosthesis fitting:**	☐ Well-fitting<br>☐ With minor misfit<br>☐ Completely ill-fitting or fractured
**11**	**Lower Denture Images**	☐ Occlusal view ☐ Fitting view

P no.	File no.	Contact no.	Date:

	**<u>Section 3: Oral Examination</u>**	
1	**Presence of Denture Stomatitis (Newton's Classification):**	☐ None ☐ Mild<br>☐ Moderate ☐ Severe
2	**Signs of Oral Fungal Infection:**	☐ None ☐ White Patches<br>☐ Redness
3	**Salivary Flow (Based on Clinical Signs):**	☐ Normal ☐ Reduced<br>☐ Dry Mouth Observed
4	**Saliva Flow Rate Measurement (modified Schirmer Test, 3 min, floor of the mouth placement):**	……mL
5	**Halitosis (Based on Breath Odour):**	☐ Yes ☐ No

<u>*Newton's Classification for Denture Stomatitis*</u>:

Mild/ Type I: is characterized by localized inflammation, often appearing as small, red dots (petechiae) on the palate.
Moderate/ Type II: involves generalized inflammation, where the entire denture-bearing area appears red and inflamed.
Severe/ Type III: is accompanied by inflammatory papillary hyperplasia, where the mucosa develops a bumpy or granular surface.
