# Supplementary material for: Denture Hygiene Knowledge, Practices, and Stated Willingness to Use a Probiotic-Based Denture Cleanser Among Removable-Prosthesis Wearers at a Saudi University Dental Hospital: A Cross-Sectional Study
Source: Healthcare (Basel). 2026 Oct 15;14(20):3466. doi: 10.3390/healthcare14203466 (PMC13704227; doi:10.3390/healthcare14203466)
Supplement: Supplementary file 1 [file healthcare-14-03466-s001.zip › Table_S1_CVI.pdf]

Table S1. Item-level content validity indices (I-CVI) for the study questionnaire, rated independently by four external expert consultants.

Item	Item (English)	Domain	I-CVI relevance	I-CVI clarity	I-CVI simplicity
1	Age	Demographics	1.00	1.00	1.00
2	Gender	Demographics	1.00	1.00	1.00
3	Nationality	Demographics	1.00	1.00	1.00
4	Marital status (+ age of youngest child)	Demographics	0.75	1.00	1.00
5	Educational level + health-related background	Demographics	1.00	1.00	1.00
6	Employment status	Demographics	1.00	1.00	1.00
7	Medical conditions and medications	Demographics	1.00	1.00	1.00
8	Antibiotics in last 3 months	Demographics/Medical history	1.00	1.00	1.00
9	Respiratory infection/sore throat in last 3 months	Demographics/Medical history	1.00	1.00	1.00
10	Smoking (current/previous/none)	Demographics/Medical history	1.00	1.00	1.00
11	Duration wearing current denture(s)	Clinical background	1.00	1.00	1.00
12	Routine dental check-up frequency	Clinical background	1.00	1.00	1.00
13	Source of denture care information	Knowledge	1.00	1.00	1.00
14	Belief that denture cleaning is essential	Knowledge	1.00	1.00	1.00
15	Awareness of specialized denture cleaning products	Knowledge	1.00	1.00	1.00
16	Heard of the oral microbiome	Knowledge	0.75	0.50	0.75
17	Belief that all oral microbes are harmful	Knowledge	1.00	0.75	1.00
18	Heard of oral probiotics	Knowledge	0.75	1.00	1.00
19	Know that yogurt contains probiotics	Knowledge	0.75	1.00	1.00
20	Frequency of wearing the denture	Practice	1.00	1.00	1.00
21	Frequency of denture cleaning	Practice	1.00	1.00	1.00
22	Use of denture adhesive	Practice	1.00	1.00	1.00
23	How dentures are cleaned (methods)	Practice	1.00	1.00	1.00
24	Previous use of oral probiotics	Practice	1.00	1.00	1.00
25	Frequency of yogurt intake	Practice	1.00	0.75	0.75
26	Oral fungal infection since wearing current denture	Problems	1.00	1.00	1.00
27	Bad breath since current denture	Problems	1.00	1.00	1.00
28	Discomfort/redness/irritation under denture	Problems	1.00	1.00	1.00
29	Willingness to try probiotic-based denture cleanser	Attitude	1.00	0.75	0.75
30	Preferred form of denture cleanser	Attitude	1.00	1.00	1.00

I-CVI = number of experts rating the item 3 or 4 on a 4-point scale, divided by four. Scale-level indices (S-CVI/Ave, the mean of the item-level values) were 0.967 for relevance, 0.958 for clarity and 0.975 for simplicity. An I-CVI of ≥ 0.78 and an S-CVI/Ave of ≥ 0.90 were considered acceptable. Seven items fell below 0.78 on at least one dimension (all 0.75, except clarity of the oral-microbiome item at 0.50); all were retained because the lower ratings reflected the technical terminology rather than a lack of relevance, and the wording of those items was simplified before use.
